# Parental perception on oral health-related quality of life and dental features of ectodermal dysplasia and isolated hypodontia in children

**DOI:** 10.1186/s12903-021-01878-5

**Published:** 2021-10-09

**Authors:** Emily Crossan, Anne C. O’Connell

**Affiliations:** Present Address: Dublin Dental University Hospital, Trinity College, Dublin, Ireland

**Keywords:** Oral health-related quality of life, Ectodermal dysplasia, Isolated hypodontia, P-CPQ, FIS, Tooth agenesis, Hypodontia, Children, Adolescents, Parents

## Abstract

**Background:**

Children missing 6 or more permanent teeth often present with complex dental care needs and significant impacts on their oral health related quality of life (OHRQoL). The most important facet in the overall care for these children is the child’s own experience, but parents primarily make the decisions regarding their child’s dental management. Understanding the parental perspective could have a positive impact on planning and provision of care for these patient groups in the future. The study compared the parental perspectives on OHRQoL impact and dental experience for children with ectodermal dysplasia (ED), severe isolated hypodontia (IH), and matched controls following assessment of their dental features.

**Design:**

A cross-sectional study of 172 children (mean age: 12.4 years old) was conducted; 86 with severe hypodontia (≥ 6 missing teeth; ED: 29; IH: 57) and 86 age and gender matched controls. The Parental-Caregiver Perceptions Questionnaire (P-CPQ), Family Impact Scale (FIS) and a supplemental questionnaire were used to gather information on parental perceptions of OHRQoL and dental experiences, respectively. Clinical examinations were used to assess and compare the dental features between children with ED, IH and their respective controls.

**Results:**

Higher scores (*p* < 0.05) were found in P-CPQ and FIS scores between the children with ED, IH and their respective controls. P-CPQ scores for males with ED had a moderate correlation with functional limitations (R_s_ = 0.576; *p* = 0.001*), oral symptoms (R_s_ = 0.444; *p* = 0.016*) and overall QoL (R_s_ = 0.499; *p* = 0.006*). The ED group reported earlier awareness of issues, the youngest attendance (3.24 years) and highest perceived number of appointments (“20 or more”; 58.6%). The mean number of missing teeth in the ED group was almost twice that of the IH group (ED: 20.17; IH: 10.68) and the median number of missing teeth (Radiographically: ED = 21; IH = 9; Clinically: ED = 11; IH = 6), was significantly greater in the ED group when compared to the IH group (*p* < 0.001*).

**Conclusion:**

Parents of children with ED and IH perceive a greater impact on QoL, for both the child and their family. Children with ED need earlier intervention and more extensive treatment than children with IH and their controls.

## Background

Children with severe hypodontia are likely to present with complex dental care needs. Hypodontia is defined as the developmental absence of one or more teeth, ranging in severity from mild to severe, including the total absence of tooth formation [1]. Severe hypodontia is generally understood as missing six or more teeth and has a prevalence of less than 1%, with reports as low as 0.25% in European populations [2]. Hypodontia can occur in the absence of other conditions, known as isolated hypodontia (IH), and may also be associated with a syndrome, Ectodermal dysplasia (ED) being the most common. ED is a diverse group of congenital conditions affecting two or more ectodermal structures, such as skin, hair, nails, teeth and sweat glands [3, 4]. Hypodontia patterns and the presence of other dental anomalies, such as malocclusions, taurodontism, conical teeth and microdontia, are significant components of the phenotypes for both ED and IH, particularly as other manifestations of ED may be very mild. These anomalies can complicate management, highlighting the importance of the entire phenotype and not just the number of missing teeth.

Severe hypodontia, both isolated and syndromic, can have a significant impact on a child’s oral health-related quality of life (OHRQoL) [5, 6]. Previous studies have shown that the predominant impacts, for both ED and IH, are related to appearance and function [5–7], and it has been suggested that there is a higher level of impact on females [5, 7]. In our experience, children with ED and IH are presenting at a young age where they may not be comfortable or able to express their concerns or wishes to the dental team. Knowledge of parental perceptions on the impact of hypodontia and its management would be very valuable for both conditions and can be captured using the Parental-Caregiver Perception Questionnaire (P-CPQ), which was specifically designed for parents. The P-CPQ forms one component of the Child Oral Health Quality of Life Questionnaire (COHQoL)[8] and also contains two global well-being questions and the Family Impact Scale (FIS). Developed as an adjunct to the Child Perception Questionnaire (CPQ), it measures the impact on the child and the family from the parent’s perspective [8]. P-CPQ was designed for parents with children aged between 6 and 14 years old, but the psychometric features have been evaluated on children from 3 years-old and upwards [9]. Parental perceptions of OHRQoL for ED and IH children have been recently reported [6, 7, 10]. These studies have used both CPQ and P-CPQ and two of the studies have [7, 10] published their mean P-CPQ scores, which were relatively high, suggesting a perceived poorer QoL. However, the parent data is not given much attention in these studies, only correlating data from the parent to that reported by the child and not to controls. Without control data for comparison, we can only estimate the perception of impact within affected populations. While the child’s perception of OHRQoL is very important, knowledge of their parents view point is also valuable, as parents are ultimately the principal decision makers and their perception of their child’s OHRQoL is likely to have the biggest influence on dental management [8, 11, 12]. To our knowledge, no previous studies have directly compared parental perceptions of OHRQoL impact for children with ED, children with IH and controls.

This study evaluated the parental perception of children’s OHRQoL using P-CPQ and FIS questionnaires and dental experience using a specifically designed questionnaire for children with ectodermal dysplasia (ED), severe isolated hypodontia (IH), and age and gender matched control children. As a secondary outcome, this study explored the dental features between children with ED and IH (≥ 6 missing permanent teeth).

## Methods

This cross-sectional study was carried out at the Dublin Dental University Hospital (DDUH) and was written in accordance with the guidelines of the ‘Strengthening the reporting of observational studies in epidemiology’ (STROBE Statement) [13]. The DDUH is a tertiary care centre, as well as a teaching hospital and the national referral centre for individuals with developmental dental conditions. Tallaght University Hospital/St. James's Hospital Joint Research Ethics Committee (JREC) granted ethical approval for this study (REC Reference 2018/07/03/2018-08 List 30 (4)/2019-10 List 37 (8)).

G*Power 3.1 software (Universität Düsseldorf version 3.1.) was used for sample size calculation based on the primary outcome of this study, where comparisons of P-CPQ mean total scores were estimated, based on Wilxocon test (matched pairs), for the ED group, IH group, and their respective controls. The mean total scores reported by Kohli et al. [7] for ED patients (mean: 35.0 ± 16.8) and Raziee et al. [10] for IH patients (mean = 24.2 ± 18.6) were used for these comparisons and estimating a difference of 40% between the ED group, IH group and their respective controls. Assuming an alpha error rate of 0.05 and power of 0.95; a minimal overall sample size of 124 would be necessary, with a minimum of 18 in the ED and the ED control groups and 44 in the IH and the IH control groups.

Between February 2019 and February 2020, patients attending the clinics of the DDUH were invited to participate in the study. Children under 18 years old and missing 6 or more permanent teeth with ED or a non-syndromic medical history were included in the study. Children were excluded if they were missing fewer than 6 permanent teeth, had a significant dental condition (e.g. Amelogenesis imperfecta) or a history of significant dental treatment (e.g. trauma, early extractions). When participants with ED and IH had been enlisted, age and gender matched healthy children with no missing teeth were recruited for the control groups from a similar population as the children with ED and IH. Informed written consent was obtained from each participant’s legal guardian.

### Data collection

#### Questionnaires

The P-CPQ portion of the COHQoL questionnaire including the global rating and Family Impact Scale (FIS), were used to gather information on parental perceptions of OHRQoL [8, 14]. The parent portion of the COHQoL contains 49 questions: there are 2 global rating questions; the P-CPQ contains 33 questions, covering four domains; oral symptoms, functional limitation, emotional well-being and social well-being and FIS has 14 items with four domains; parental and family activities, parental emotions, family conflict, and financial burden [8, 14]. The responses dictate the frequency of each issue; never = 0, once/twice = 1, sometimes = 2, often = 3, and everyday/almost every day = 4. A don’t know option is also available [8, 14]. Each domain is scored individually and then totalled to give an indication of the OHRQL impact. Overall, a high score is indicative of a poorer QoL.

A specifically designed questionnaire (10 questions; Table 1) was used to collect information not gathered by the P-CPQ and FIS questionnaires. This questionnaire was piloted to ensure ease of understanding among the general population and accurate collection of the intended data [15]. Parents self-completed all questionnaires on Survey Monkey ™ on a password protected iPad while their child was being examined. The participants’ unique identifying code, age, gender and the relationship of the guardian (e.g. mother or father) were also recorded.

**Table 1:** Specifically designed questionnaire

1	What age was your child at their very first dental visit:
2	What first motivated you to bring them to the dentist?
3	Where did your child attend the dentist?
4	What concerned you most about your child’s teeth? Please Rank from 1–6 (1 being of most concern and 6 of least concern): Function; Speech; Reaction of other children; Reaction of other parents; Your child’s reaction (self-conscious); How the teeth look
5	In your opinion, has your child ever been self-conscious about their teeth? If yes, please specify at what age?
6	How many dental visits has your child had? Response options: Less than 5 visits/5–10 visits/10–20 visits/20 or more visits
7	Was your child cooperative for dental visits? If you answered ‘No’, Why do you think your child was not cooperative?
8	Where did you get information about your child’s dental condition? Did you receive enough information?
9	Please specify what additional Information needed:
10	If you could start from the beginning again, what would you change?

#### Examinations

A comprehensive dental assessment, including medical and dental history was provided for each participant in a dental clinic. Radiographs were available from the treating clinician and clinical photographs were taken. The diagnosis of hypodontia was made radiographically by the absence of a tooth, tooth bud or calcification and a negative extraction history (excluding third molars). Dental caries was recorded if cavitation into dentine was detected in either a primary or permanent tooth [16]. Infraocclusion was identified clinically and recorded as mild, moderate or severe for both primary and permanent teeth [17]. Taurodontism was recorded if a fully developed permanent multi-rooted (molar) tooth displayed an enlarged pulp chamber extending apically on the OPG [18]. To increase reliability between examiners the first 10 individuals were examined separately by each of the examiners (EC and SA). Any differences were discussed until a consensus was reached between examiners. Following this, 10 random participants were scored by each examiner and then compared in a Kappa analysis.

Tooth site absence (TSA) analysis as described by Raziee et al. was used to record the presence of a clinically edentulous site instead of radiographical absence of a tooth, to represent a site that contained neither a primary nor a permanent tooth [10]. This approach provides an accurate representation of the actual number of teeth present in the patient’s mouth at the time of examination, irrespective of whether the tooth was primary or permanent. The tooth agenesis code (TAC) was developed by Van Wijk and Tan in 2006 to analyse hypodontia patterns using a binary system [19]. Using an excel worksheet registering the clinical and radiographical diagnosis of hypodontia in each patient, the TAC data analysis tool generates the hypodontia patterns present in a sample and the frequency of each pattern by assigning a unique hypodontia code to each pattern [19]. The clinical examination, radiograph and clinical photographs were used to identify the location of each missing tooth for TAC and TSA analysis.

### Data analysis

Anonymised data tabulation was performed using Excel files (Microsoft® Excel for Mac Version 16.16.23). Statistical analyses were performed using Statistical Package for the Social Sciences (SPSS® for Mac, Version 26.0, SPSS Inc., Chicago, IL, USA). For quantitative variables, Kolgomorov-Smirnov testing was used to establish data distribution.

#### Primary outcome

The global, P-CPQ and FIS scores were compared between the ED/IH groups and their respective control group using the Wilcoxon test.

#### Secondary outcomes

The secondary outcomes of this study included comparisons between the ED and IH groups for global, P-CPQ and FIS scores. Moreover, answers from the specifically designed questionnaire and dental features were compared between the ED and IH groups and also between their respective controls. The paired and unpaired non-parametric analyses of the independent quantitative variables were conducted using Wilcoxon signed-rank test and Mann–Whitney U test, respectively. Spearman correlations were carried out for variables related to P-CPQ. Chi-square, Fisher’s Exact and McNemar analyses were performed, for paired and unpaired qualitative variables, respectively. The Bonferroni corrected critical *p* value of 0.017 for statistical significance was considered to manage multiple comparisons within the data. In analysis of the clinical features between ED and IH, SPSS (V26.0) was used to generate cross-tabulations from which raw Odds Ratios and their confidence intervals were calculated, which considered a confidence level of 95%, α = 5% and a critical value of 1.96 and the reference groups were altered to allow for all Odds Ratio values to be represented as OR > 1 for easier interpretation.

Descriptive TSA analysis were performed for the data denoting the clinical presence or absence of each tooth for each tooth site, excluding 3^rd^ molars. Data denoting the radiographical presence or absence of each tooth, excluding 3^rd^ molars, were uploaded to the TAC data analysis tool website, (http://www.toothagenesiscode.com/) for TAC analysis [19].

## Results

**Fig. 1:**
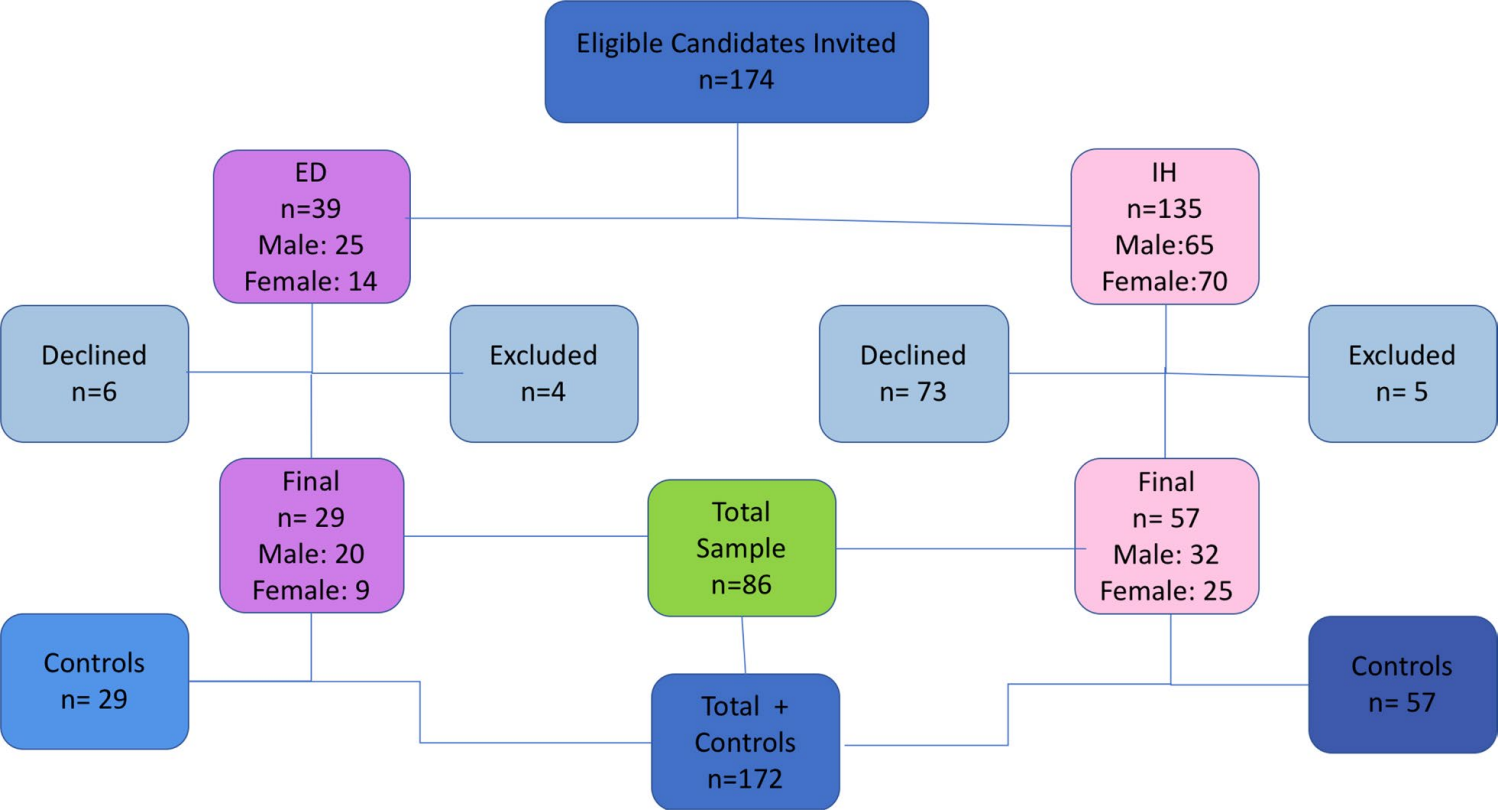
Distribution of candidates between ED, IH and control groups. n = number of individuals

One hundred and seventy-four patients were invited to participate in the study. The majority had IH (135) however 73 patients declined to participate and 5 were excluded, as they were missing fewer than 6 teeth. Fifty-seven individuals with IH were included in the study (25 females and 32 males, with a mean age of 13.4 years). Thirty-nine individuals with ED were invited to participate, 6 declined to participate and 4 were excluded as they were missing fewer than 6 teeth. The remaining 29 were included in the study, (9 females and 20 males, with a mean age of 10.5 years). For most of the individuals who declined to participate they did not provide a reason and the remaining did not wish to attend for a separate appointment. The total sample with complete data was 86, aged between 4 and 18-years old and is summarised in Fig. 1. Age and gender-matched controls were recruited for each participant, bringing the total sample to 172. Most participants were Caucasian (83.1%) and the majority of all participants were accompanied by their mothers (72.1%).

### P-CPQ questionnaire results

The majority of all questionnaires were completed by mothers (72.1%). The global rating, P-CPQ and FIS data were not normally distributed and subsequently nonparametric tests were utilised. Both the ED and IH groups scored higher than their controls in the global rating (ED: 0.005; IH: 0.015), suggesting a poorer overall perception of QoL for the ED and IH groups. The mean P-CPQ scores for all groups are depicted in Table 2 and the mean FIS scores are detailed in Table 3. Greater scores (*p* < 0.05) were noted in all P-CPQ and FIS scores from the ED group compared to their matched controls (Tables 2, 3). The IH group also had greater P-CPQ scores than their matched controls, particularly in the overall score (*p* < 0.001), the emotional well-being domain (*p* = 0.008) and the social well-being domain (*p* = 0.006) (Table 2). Their FIS scores were also all greater than their controls and greater differences were noted in the parental emotional well-being domain of the FIS (*p* < 0.001) and the overall total FIS score (*p* = 0.009) (Table 3), suggesting a greater impact is perceived by parents for participants with both ED and IH when compared to controls. In an unpaired analysis of the ED group compared to the IH group, the ED mean scores were higher in all domains and were greater for functional limitations (*p* < 0.001**) (Table 4).

**Table 2:** Descriptive paired analysis of mean P-CPQ scores for all groups

Variables	Oral symptoms Mean (SD)	Functional limitations Mean (SD)	Emotional well-being Mean (SD)	Social well-being Mean (SD)	Total P-CPQ Mean (SD)
*ED group*					
Control (29)	3.07 (2.90)	3.28 (5.30)	1.86 (3.56)	2.41 (4.71)	10.62 (14.28)
ED (29)	5.45 (4.63)	11.79 (7.71)	8.0 (9.08)	7.93 (9.76)	33.17 (28.46)
Wilcoxon	*p* = 0.025*	*p* < 0.001**	*p* = 0.002**	*p* = 0.002**	*p* < 0.001**
*IH group*					
Control (57)	4.02 (3.43)	3.49 (4.51)	3.04 (4.26)	3.75 (4.97)	14.30 (14.47)
IH (57)	4.18 (3.80)	5.39 (6.07)	7.02 (8.31)	6.74 (7.30)	28.89 (22.37)
Wilcoxon	*p* = 0.867	*p* = 0.123	*p* = 0.008**	*p* = 0.006**	*p* < 0.001**

*p < 0.05; Bonferroni corrected critical ** *p* < 0.017; (SD) = Standard Deviation

**Table 3:** Descriptive paired analysis of mean FIS scores for all groups

Variables	Activities Mean (SD)	Emotional Mean (SD)	Conflict Mean (SD)	Financial Mean (SD)	Total FIS Mean (SD)
*ED group*					
Control (29)	2.72 (3.52)	0.52 (1.30)	0.72 (1.58)	0.17 (0.468)	4.14 (5.91)
ED (29)	6.07 (4.94)	3.41 (2.53)	2.62 (2.95)	0.83 (1.00)	12.93 (10.38)
Wilcoxon	*p* = 0.006**	*p* < 0.001**	*p* = 0.006**	*p* = 0.004**	*p* < 0.001**
*IH group*					
Control (57)	2.44 (2.57)	0.35 (0.61)	1.11 (2.09)	0.37 (0.79)	4.16 (5.10)
IH (57)	3.89 (3.77)	1.49 (1.79)	1.56 (2.11)	0.44 (0.78)	7.39 (6.85)
Wilcoxon	*p* = 0.053	*p* < 0.001**	*p* = 0.173	*p* = 0.640	*p* = 0.009**

*p < 0.05; ** *p* < 0.017

**Table 4:** Descriptive unpaired analysis of mean P-CPQ scores

Variables	Oral symptoms Mean (SD)	Functional limitations Mean (SD)	Emotional Well-being Mean (SD)	Social well-being Mean (SD)	Total P-CPQ Mean (SD)
*ED and IH groups*					
IH (57)	4.18 (3.80)	5.39 (6.07)	7.02 (8.31)	6.74 (7.30)	28.89 (22.37)
ED (29)	5.45 (4.63)	11.79 (7.71)	8.0 (9.08)	7.93 (9.76)	33.17 (28.46)
Mann Whiney U	*p* = 0.143	*p* < 0.001**	*p* = 0.561	*p* = 0.822	*p* = 0.528

*p < 0.05; Bonferroni corrected critical **p < 0.017; (SD) = Standard Deviation

**Fig. 2:**
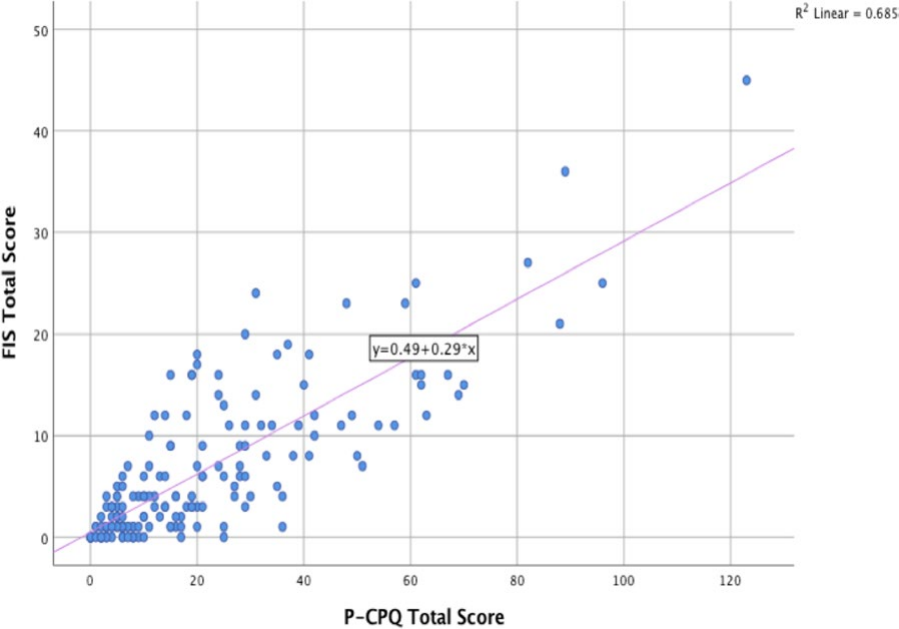
Dot-plot graph displaying strong positive correlation between P-CPQ and FIS scores. Y axis = FIS scores; X axis = P-CPQ scores; for entire sample

There were more males than females, with 9 females and 20 males in the ED group; and 25 females and 32 males in the IH group. Spearman correlation revealed a moderate correlation for P-CPQ scores and gender within the ED group, indicating a greater impact for males with ED, when compared to females with ED, on functional limitations (R_s_ = 0.576; *p* = 0.001*), oral symptoms (R_s_ = 0.444; *p* = 0.016*) and overall QoL (R_s_ = 0.499; *p* = 0.006*). For the IH group, there were weak correlations between being female and all domains except for functional limitations. However, none of these correlations reached statistical significance. Our study included a wide range of age groups (4–18-years old). A moderate correlation of Rs = 0.449 (*p* < 0.001*) was found for the IH group between increasing age and oral symptoms. No other significant correlations were found between age and P-CPQ scores. For all the groups the scores from the P-CPQ and FIS overall were strongly correlated, (Rs = 0.789; < 0.001*) showing consistent results between the instruments, as depicted in Fig. 2.

### “Don’t know” (DK) responses in P-CPQ

Overall, parents used the “Don’t Know” (DK) response to answer 4.4% of the P-CPQ and FIS questions. This increased to 5.6% when FIS responses were excluded. Both the ED and IH groups had higher numbers of DK responses, 7.5% and 9.7% respectively, when compared to controls (2.2%).

## Specifically designed questionnaire

**Fig. 3:**
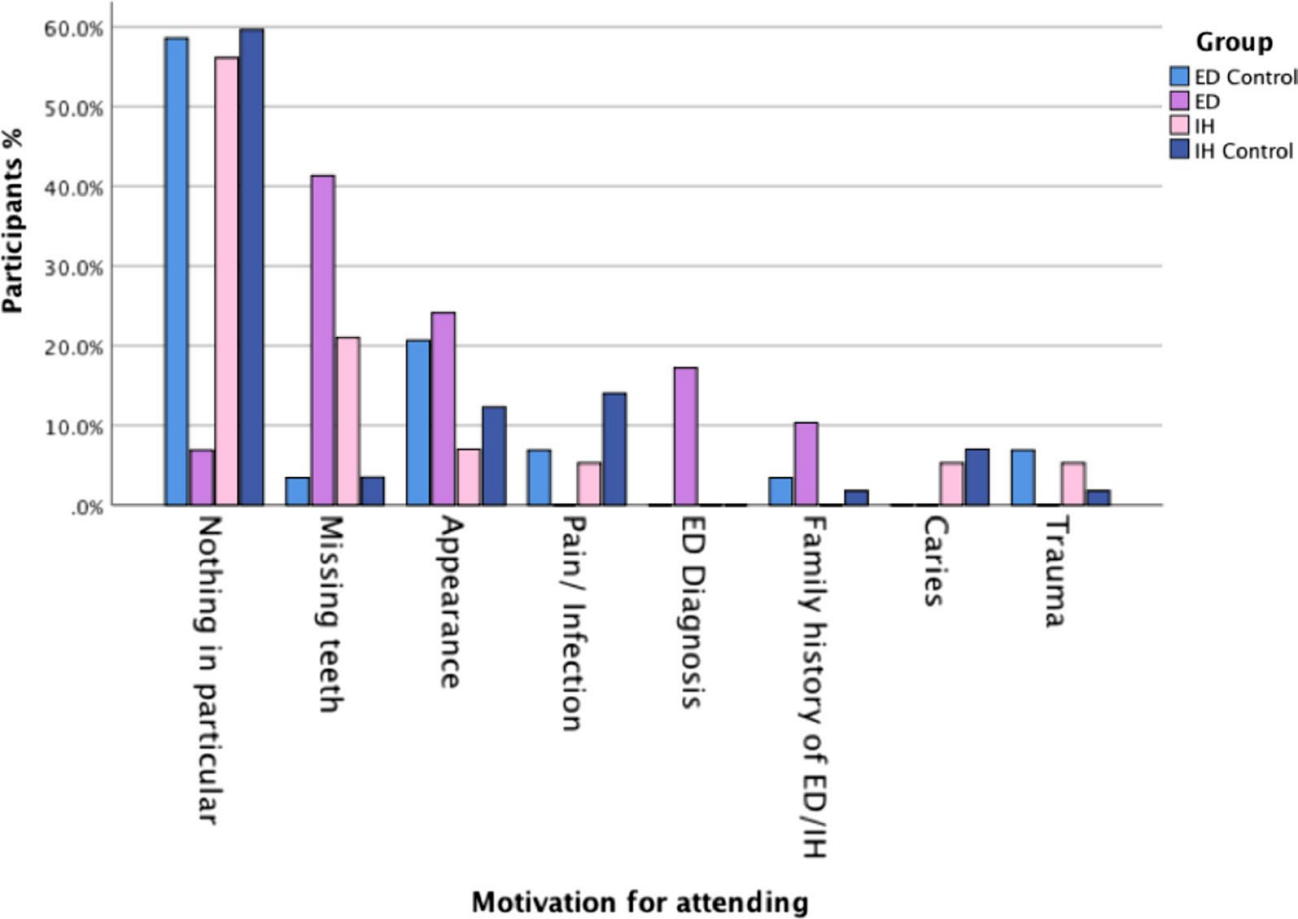
Bar chart comparing the motivation for the child’s first appointment to the dentist for the four groups: ED control, ED, IH, IH Control. Y axis = percentage of participants; X axis = motivation category (Routine, missing teeth, appearance, pain/infection, ED diagnosis, positive family history of ED/IH, caries, trauma)

The specifically designed questionnaire (Table 1) revealed that the mean age for a child’s first dental visit was lowest in the ED group, 3.24 years (SD ± 2.23) (IH group: 5.19 years, SD ± 2.27; IH control group: 6.19 years, SD ± 2.63; ED control group: 4.79 years, SD ± 2.19). The main motivation for the first dental visit in the ED group was ‘Missing teeth’ (41.4%), whereas ‘nothing in particular/general check-up’ motivated the first visit for both the IH and control groups (IH: 56.1%; IH control: 59.6%; ED Control: 58.6%) (Fig. 3). Both the IH and control groups ranked appearance (Q4:‘How the teeth look’) as the most important feature, whereas the ED group ranked ‘Function’ as the most important. All groups agreed that the ‘reaction of other parents’ was the least important factor. More parents in the ED and IH groups reported their child was self-conscious about their teeth/mouth than parents in the control groups, but this was only significant for the ED group (ED vs ED controls: *p* = 0.018*; IH vs IH controls: *p* = 0.703). Parents reported that their children’s concern about their teeth presented at a median age of 6 years-old for the ED group, age 10 years-old for the IH group and ages 9 years-old and 10 years-old for the respective control groups. The majority of parents in all groups reported their children were cooperative for dental treatment. For those who reported a lack of cooperation, the main reason given in all groups was dental anxiety, followed by being ‘too young’ for dental treatment. The majority of all groups reported the dentist as their main source of information about their child’s mouth/teeth. The ED group reported the highest number of appointments (20 or more dental visits; 58.6%), compared with the IH (26.3%) and the control groups (ED controls: 17.2%; IH controls: 15.8%). Parents from the ED and IH groups reported the main information deficits were related to treatment timing and lack of explanation of the condition itself. Finally, when asked ‘if they could start the treatment over again, what would they change’, the majority in all groups reported they would ‘not change anything’, followed by ‘start treatment earlier’.

### Clinical examination

An inter-examiner analysis showed good agreement between examiners with Kappa scores greater than 0.8 for all of the examination variables.

### Hypodontia

In the ED group there was an average of 20.17 (s.d. = 5.85; range 6–28) missing permanent teeth. The lower central incisors were missing for all ED participants (100%) and the maxillary lateral incisors were missing in all but one participant (96.6%). All tooth-types were affected by hypodontia in the ED group, with the maxillary central incisors being the least common (32.8%). Approximately 73% of second molars and 60.3% of canines were missing in the ED group. In the IH group there was an average of 10.68 (s.d. = 4.41; range 6–23) missing permanent teeth. Approximately 77% of second premolars were absent in the IH group, followed by the maxillary first premolars (60.5%) and the maxillary lateral incisors (52.6%). The maxillary central incisors were not missing in any participants in the IH group. Almost 29% of second molars and 26% of canines were missing in the IH group. Table 5 summarises the variation in location and prevalence of missing teeth between children with ED and IH. No significant differences were observed between the left and right sides of the mouth.

**Table 5:** Distribution of missing permanent teeth in IH and ED groups

Tooth	IH Maxillary n(% n/114 × 100)	ED Maxillary n(%n/58 × 100)	IH Mandibular n(%n/114 × 100)	ED Mandibular n(%n/58 × 100)	IH Total n(%n/228 × 100)	ED Total n(%n/116 × 100)
Central incisor	0 (0%)	19 (32.8%)	45 (39.5%)	58 (100%)	45 (19.7%)	77 (66.4%)
Lateral incisor	60 (52.6%)	56 (96.6%)	34 (29.8%)	51 (87.9%)	94 (41.2%)	107 (92.2%)
Canine	39 (34.2%)	36 (62.1%)	20 (17.5%)	34 (58.6%)	59 (25.9%)	70 (60.3%)
First premolar	69 (60.5%)	47 (81.0%)	45 (39.5%)	44 (75.9%)	114 (50%)	91 (78.4%)
Second premolar	90 (78.9%)	49 (84.5%)	86 (75.4%)	46 (79.3%)	176 (77.2%)	95 (81.9%)
First molar	34 (29.8%)	31 (53.4%)	21 (18.4%)	29 (50.0%)	55 (24.1%)	60 (51.7%)
Second molar	28 (24.6%)	45 (77.6%)	38 (33.3%)	40 (69.0%)	66 (28.9%)	85 (73.3%)

### Tooth-site absences (TSA)

**Fig. 4:**
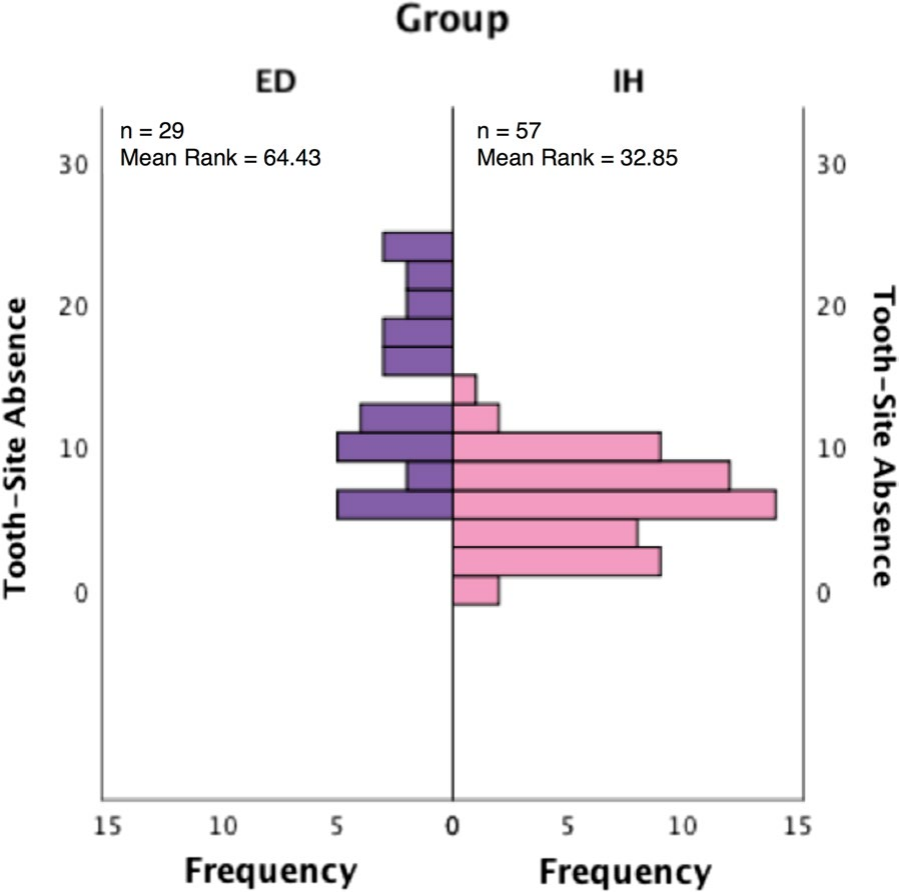
Bar chart comparing the frequency of the clinical tooth-site absences in the ED and IH groups. n = number of individuals. Y axis = number of clinical tooth-site absences (0–28); X axis = frequency (ie number of patients with that number of clinically absent tooth units)

The median number of radiographically missing teeth (ED = 21; IH = 9), the median number of TSAs (clinically missing; ED = 11; IH = 6), and the median number of anterior missing teeth (ED = 9; IH = 2) were all greater in the ED group when compared to the IH group (*p* < 0.001*). The distribution of TSA is depicted in Fig. 4 and clearly demonstrates the disparity between the groups.

### TAC

The IH group contained 37 unique TAC patterns in the maxilla, 43 unique patterns in the mandible and 54 unique patterns in the entire dentition overall. The ED group contained 25 unique patterns in the maxilla, 20 unique patterns in the mandible and 29 unique patterns in the entire dentition overall. There were no common patterns between ED and IH groups when comparing the entire dentitions.

### Other clinical findings

The ED group had a much lower prevalence of caries and hypomineralisation and far fewer restorations (due to caries) when compared to both the ED controls and the IH group (Table 6). In the current sample, the IH group were 3 times more likely than the ED group to have caries (OR: 3.00; CI: 1.06–8.47) (Table 7). In contrast, the ED group showed a higher prevalence of taurodontism (OR: 79.33; CI: 9.61–655.01), conical morphology (OR: 56.45; CI: 11.63–274.04) and aesthetic restorations (OR: 13.57; CI: 4.51–40.81) when compared to the IH group (Table 7). The prevalence of infraocclusion was greater in both the ED and IH groups than in the control groups (Table 6). As part of their dental history participants were asked if they frequently had a ‘dry mouth’. Almost half (48%) of the ED group reported xerostomia, with only 3.5% in the IH group and only 1.8% in the IH control group (0% in the ED control group) reporting xerostomia. They were also asked, in their dental history, about the number of dentures they have had made for them. The ED group were almost 13 times (OR:12.80; CI: 3.96–41.40) more likely to wear a denture than the IH group, with 55.2% (n = 16) of the ED group wearing a denture compared to 8.8% (n = 5) of the IH group and 0% in the control groups (Table 7). Of those wearing dentures (n = 21), the median age at first denture for the ED group was approximately 4 years of age compared to 14 years in the IH group. The ED group had a median of three dentures overall compared to just one in the IH group.

**Table 6:** Comparison of clinical characteristics in ED, IH and their control groups

Assessment	ED (29) n (%)	ED (29) controls n (%)		IH (57) n (%)	IH controls (57) n (%)	Total sample (172) n (%)
*Caries (both dentitions)*						
**No**	23 (79.3%)		14 (48.3%)	32 (56.1%)	36 (63.2%)	105 (61%)
**Yes**	6 (20.7%)		15 (51.7%)	25 (43.9%)	21 (36.8%)	67 (39%)
Fisher’s exact	*p* = 0.014**			*p* = 0.445		–
McNemar	*p* = 0.035*			*p* = 0.556		–
*Restorations (fillings due to caries) (both dentitions)*						
No	21 (72.4%)		15 (51.7%)	29 (50.9%)	28 (49.1%)	93 (54.1%)
Yes	8 (27.6%)		14 (48.3%)	28 (49.1%)	29 (50.9%)	79 (45.9%)
Fisher’s Exact	*p* = 0.104			*p* = 0.851		–
McNemar	*p* = 0.180			*p* = 1.000		–
*Microdont (both dentitions)*						
No	11 (37.9%)		28 (96.6%)	22 (38.6%)	51 (89.5%)	112 (65.1%)
Yes	18 (62.1%)		1 (3.4%)	35 (61.4%)	6 (10.5%)	60 (34.5%)
Fisher’s exact	*p* < 0.001**			*p* < 0.001**		–
McNemar	*p* < 0.001**			*p* < 0.001**		–
*Infraocclusion (only noted in 1° dentition)*						
No	21 (72.4%)		28 (96.6%)	36 (63.2%)	53 (93.0%)	138 (80.2%)
Yes	8 (27.6%)		1 (3.4%)	21 (36.8%)	4 (7%)	34 (19.8%)
Fisher’s exact	*p* = 0.025*			*p* < 0.001**		–
McNemar	*p* = 0.039*			*p* < 0.001**		–
*Hypomineralisation (both dentitions)*						
No	26 (89.7%)		17 (58.6%)	42 (73.7%)	43 (75.4%)	128 (74.4%)
Yes	3 (10.3%)		12 (41.4%)	15 (26.3%)	14 (24.6%)	44 (25.6%)
Fisher’s exact	*p* = 0.007**			*p* = 0.830		–
McNemar	*p* = 0.022*			*p* = 1.000		–
*Hypoplasia (both dentitions)*						
No	28 (96.6%)		29 (100%)	55 (96.5%)	56 (98.2%)	168 (97.7%)
Yes	1 (3.4%)		0 (0%)	2 (3.5%)	1 (1.8%)	12 (7%)
Fisher’s exact	*p* = 1.000			*p* = 1.000		–
McNemar	*p* = 1.000			*p* = 1.000		–
*Taurodontism (2*^*o*^* molar teeth only)*						
No	12 (41.4%)		24 (82.8%)	56 (98.2%)	50 (87.7%)	142 (82.6%)
Yes	17 (58.6%)		5 (17.2%)	1 (1.8%)	7 (12.3%)	30 (17.4%)
Fisher’s exact	*p* = 0.001**			*p* = 0.061		–
McNemar	*p* = 0.004*			*p* = 0.070		–

Significant *p values < 0.05 and the Bonferroni corrected critical ** *p* values < 0.017

n = Number; (%) = Percentage; 1° = Primary; 2^o^ = Secondary; Both = Primary and Secondary

**Table 7:** Comparison of clinical characteristics in ED and IH groups

Assessment	ED (29) n (%)	IH (57) n (%)	*p* value	OR 95% CI	OR (Ref)
*Caries (both dentitions)*					
No	23 (79.3%)	32 (56.1%)			
Yes	6 (20.7%)	25 (43.9%)	0.034*	3.00 (1.06–8.47)	ED
*Restorations (both dentitions)*					
No	21 (72.4%)	29 (50.9%)			
Yes	8 (27.6%)	28 (49.1%)	0.056	2.53 (0.97–6.66)	ED
*Microdont (both dentitions)*					
No	11 (37.9%)	22 (38.6%)			
Yes	18 (62.1%)	35 (61.4%)	0.952	1.03 (0.41–2.58)	IH
*Conical (both dentitions)*					
No	2 (6.9%)	46 (80.7%)			
Yes	27 (93.1%)	11 (19.3%)	< 0.001**	56.45 (11.63–274.04)	IH
*Aesthetic restorations (both dentitions)*					
No	10 (34.5%)	50 (87.7%)			
Yes	19 (65.5%)	7 (12.3%)	< 0.001**	13.57 (4.51–40.81)	IH
*Infraoccluded (only noted in 1° teeth)*					
No	21 (72.4%)	36 (63.2%)			
Yes	8 (27.6%)	21 (36.8%)	0.391	1.53 (0.58–4.01)	ED
*Hypomineralised (both dentitions)*					
No	26 (89.7%)	42 (73.7%)			
Yes	3 (10.3%)	15 (26.3%)	0.085	3.01 (0.82–11.73)	ED
*Taurodontism (2*^*o*^* molar teeth only)*					
No	12 (41.4%)	56 (98.2%)			
Yes	17 (58.6%)	1 (1.8%)	< 0.001**	79.33 (9.61–655.01)	IH
*Wearing denture*					
No	13 (44.8%)	52 (91.2%)			
Yes	16 (55.2%)	5 (8.8%)	< 0.001**	12.80 (3.96–41.40)	IH

Fisher’s Exact *p values < 0.05 and the Bonferroni corrected critical ** *p* values < 0.017

OR = Odds Ratio; CI = Confidence Interval

n = Number; (%) = Percentage; 1° = Primary; 2^o^ = Secondary; Both = Primary and Secondary

## Discussion

This cross-sectional study included 86 sets of participants and parents (57 children with IH and 29 children with ED). Each participant had a control, matched for age and gender, who were also recruited from DDUH clinics, allowing participants to be compared with patients of a similar background of hospital-based care. The DDUH is a national tertiary referral centre, as well as a teaching hospital and accepts individuals with various developmental dental conditions for dental management at no financial cost to the family. In this study, the majority of the participants were Caucasian at 83%, representative of the population of Dublin in 2020, (90% of Caucasian), and similar to the national 2016 Irish census (82.2% Caucasian) [20, 21].

The current literature reports a substantial negative impact for severe hypodontia, both syndromic and isolated, regardless of the instrument used to assess OHRQoL [5, 6, 22–25]. The findings from our study are in agreement with the literature, parents from both the ED and IH groups reported a high impact on QoL. The P-CPQ questionnaire has been validated for many different oral conditions and was specifically designed to investigate the parental perceptions of the impact of oral conditions on their children and outperformed a hypodontia-specific QoL instrument in a recent systematic review [9]. Recent studies have investigated the parental perceptions of OHRQoL for ED and IH groups [6, 7, 10] and are compared in Table 8. Each of the studies report high P-CPQ scores from parents of children with hypodontia, but the parent data is mostly reported in correlation to the child’s data and not to data from a control group [6, 7, 10]. It is difficult to compare previous studies due to the numerous differences in study design, data management and analysis. Raziee et al. conducted a study with 35 children with IH (all missing ≥ 6 permanent teeth) with no controls and a mean age of 12.4 years similar to this study (13.4 years)[10]. When analysing their P-CPQ data they combined the oral symptoms and functional limitation domains and the emotional and social well-being domains and appear to have renamed the FIS as social well-being, making comparison difficult and casting some doubt on their intra-study analysis between their CPQ and P-CPQ data (Mean scores available in Table 8). Kotecha et al. conducted a study with 86 children with IH, 43 of whom were missing ≥ 6 permanent teeth, with a mean age of 12.5 years and 30 controls [6]. P-CPQ data was not published but a moderate correlation between parental and the child data was reported. Kohli et al. included only children with ED (n = 35) and collected data in 2003 and then again in 2007 [7]. In 2003, the COHQoL was still in its developmental stages and the version used by Kohli et al. for their initial data collection in 2003 was missing two questions from the emotional well-being domain. For continuity, these two additional questions were also not included in the 2007 data collection. Similar to Raziee et al., only their mean overall scores were only compared to their child data (Mean scores available in Table 8). Kohli et al. showed that functional impacts increased with age for ED individuals [7]. Interestingly in our study, only oral symptoms scores increased with age for the IH group, with no other correlations for any of the groups reaching statistical significance. Kohli et al. [7] found a significantly greater impact on emotional well-being for female participants with ED in their study. However, this study found that males had a moderate correlation (R_s_ = 0.576; *p* = 0.001*) with functional limitations, oral symptoms (R_s_ = 0.444; *p* = 0.016*) and overall QoL (R_s_ = 0.499; *p* = 0.006*) and no statistically significant correlation with emotional well-being. The gender inequality (n = 20 males, 69%) in our ED sample is a limitation of this study, but not unexpected given the nature of the genetic inheritance of ED and the gender distribution is similar to that of Kohli et al. [7] (Kohli et al.: 63%, n = 22 males; This study: 69%, n = 20 males). However, it is also important to remember that our findings reflect only the parental perceptions of QoL impact versus the child’s perspective in Kohli et al. [7] and this may account for these differences. Our findings highlight the need for further research.

**Table 8:** Mean P-CPQ scores in comparison with those reported in other studies

Study	Group	Sample (n)	Domain 1	Domain 2	Domain 3	Domain 4	Total
			Mean ± SD				
Kohli et al. 2011	ED	35	7.9 ± 4.3	9.8 ± 5.5	8.4 ± 5.1	8.9 ± 7.7	35.0 ± 16.8
Kotecha et al. 2013	IH	86 (43 with severe IH and 43 with mild IH)	Not available/Not published				
Raziee et al. 2019 (merged scores for Domains 1&2 and 3&4)	IH	35	8.6 ± 6.2		10.0 ± 9.6		24.2 ± 18.6
Current study	ED	29	5.5 ± 4.6	11.8 ± 7.7	8.0 ± 9.1	7.9 ± 9.8	33.2 ± 28.5
	IH	57	4.2 ± 3.8	5.4 ± 6.1	7.0 ± 8.3	6.7 ± 7.3	28.9 ± 22.4

FIS provides an indication of the overall impact on the family and FIS scores are strongly correlated with P-CPQ scores (Fig. 4), meaning that parents who reported a higher impact on QoL in the P-CPQ were also likely to report a higher impact on their family and vice versa. It’s unclear if Kotecha et al. [6] or Kohli et al. [7] included FIS in their studies as it is not mentioned in either paper. Raziee et al. [10] did include the FIS portion of the questionnaire, however appear to have relabelled the FIS as the social well-being domain in P-CPQ. If their social well-being domain is the FIS portion of the questionnaire, then their reported mean overall FIS score (5.6, SD: 6.7) is greater than our control mean overall FIS score (4.16, SD: 5.10) but less than our IH mean overall FIS score (7.39, SD: 6.85). This suggests that Raziee et al. [10] also found a negative impact on families, albeit to a lesser degree. This study highlights the significant negative impact both ED and IH have on the child’s family, particularly for those with ED, and perhaps the need for clinicians to be mindful of this impact when providing care for these children.

The specifically designed questionnaire provided additional insight and it highlighted the importance of clear communication between the clinician, the patient and their parents. This questionnaire was designed to capture valuable clinical information not included in other questionnaires and revealed that children with ED present at a much younger age (ED: 3.24 years, SD ± 2.23) compared to all other groups, and this was primarily prompted by parents concern over ‘Missing teeth’ (41.4%). Our results showed that parents of children with ED value function over aesthetics and vice versa for the parents of children with IH. Parents from both groups felt their children were self-conscious about their teeth/mouth. Interestingly, parents of children with ED felt that their children became self-conscious about their teeth at a younger age (6 years-old) than the IH (10 years-old) and the control groups (9 years-old and 10 years-old). The ED group also reported the highest number of appointments, compared with the IH and the control groups. From our results the dental ‘burden’ clearly seems to be considerably greater for children with ED.

All participants in the ED and IH groups were all classified as having severe hypodontia, but participants in the ED group were missing twice as many teeth than the children in the IH group. The maxillary central incisors were the least commonly missing teeth in both groups, never missing in the IH group but 32.8% were absent in the ED group. Dhamo et al. proposed that missing second permanent molars, the presence of abnormally-shaped incisors and canines and a 1 year delayed dental development of the teeth present could potentially be one of the phenotypic indicators to discriminate ED from severe IH [26]. Approximately 73% of second molars were missing in the ED group compared to 29% in the IH group. However in our sample, similarly large differences also existed between the mandibular central (100% vs 39.5%) and lateral incisors (Max: 96.6% vs 52.6%; Mand: 87.9% vs 29.8%), canines (Max: 62.1% vs 34.2%; Mand: 58.6% vs 17.5%), and first molars (Max: 53.4% vs 29.8%; Mand: 50.0% vs 18.4%), in both groups. Schalk-van der Weide et al. broadens the criteria and suggests that ED should be considered if the most stable teeth (Max central incisors, second molars and canines) are missing, or in those missing a large number of teeth, which may be more fitting than Dhamo’s hypothesis [27]. Our study suggests that a combination of these theories should be considered and subsequently prompt further investigation; including the presence of conical teeth, 1-year delayed development, hypodontia of the more “stable teeth”, particularly the maxillary central incisor and potentially the mandibular central incisor, missing large numbers of teeth (particularly greater than 10) and taurodontism.

In keeping with the current literature, the ED group showed the highest prevalence of taurodontism, conical morphology, and microdontic morphology [26, 28]. Taurodontism was present in 58% of the ED group in this study, which is comparable with a previous report [28]. Within the IH group, only 1 participant (1.8%) presented with taurodontism, considerably lower than reported previously (35–52%) [29, 30]. Conical tooth-morphology was present in 93% of the ED group compared to 19% in the IH group. Dhamo et al. had a similar finding of 17.1% in the IH group but reported a much lower prevalence of conical teeth (63.6%) in the ED group [26].

TAC has been reported to allow easy comparison of hypodontia patterns [19]. The TAC tool generates codes to represent the hypodontia pattern using a binary system [19]. Although the concept and potential of TAC is interesting, ultimately for this study, TAC provided no additional information or benefit, as it produced 29 unique patterns for the 29 participants with ED and 54 unique patterns for the 57 participants with IH. The inability of the TAC tool to allow differentiation between groups in a single sample, in particular, is a major drawback of the index.

The number of tooth site absences, and missing anterior teeth was much greater in the ED compared to the IH group. It is not surprising therefore that there was an increased prevalence of denture use in the ED group (55.2% of ED group; 8.8% of IH group; 0% of control group). For the ED group, the increased number of TSA’s meant that they were provided with their first dentures much earlier (approx. 4-years old compared to 14-years old in the IH group). This difference of 10 years results in a considerable difference in the required intervention, with those in the ED group having a median of three dentures compared to just one in the IH group. The ED group had the lowest prevalence of caries and restorations (due to caries). It was surprising that despite approximately 50% of the ED group reporting symptoms of a dry mouth and also wearing  a denture, that the potential lack of saliva and presence of a denture did not increase a patient’s caries risk. However, all of the clinical features analysis between ED and IH are secondary analysis, and therefore, results should be interpreted with caution, given the large variation of clinical characteristics shown.

This study focused on the parent’s perspectives as this data has been overlooked in previous studies. Knowing where parents place value and where and when they perceive issues, such as self-consciousness in their child, is a key component to understanding a parent’s perspective. An understanding of parents’ viewpoints and areas of concern is fundamental to the successful planning and provision of care for these patient groups. A prospective study following patients and parents and incorporating questionnaires throughout their life journey from first assessment through adolescence and into adulthood, would provide great insight and could be very valuable for future research and inform decision making in the area of service provision.

Dental treatment for children with severe hypodontia is currently subsidised by public funds in Ireland, reducing any effect of financial burden and should be considered when interpreting this data. There is also a risk of participation bias in this study, particularly in the IH group, due to the high numbers of patients who declined to participate in the study. Another recognised limitation may be that for many of the participants, treatment is mainly carried out by a local dentist with oversight being provided in the DDUH. This may also be considered a strength of the study, in that the participants involved represented the national picture and not just one area, strengthening the generalisability of the data. This research provides insight into the parental perceptions of OHRQoL for children with severe hypodontia related to ED and IH, which is especially relevant for dentists providing care for children with severe hypodontia.

## Conclusions

This study highlights the valuable information that can be gained from including parental perceptions of OHRQoL for children with ED and IH, demonstrating that parents of children with ED and IH (missing ≥ 6 permanent teeth) perceive a significantly greater impact on QoL compared to unaffected children, for both the child and their family. In particular children with ED, have a greater perceived impact on function and undergo earlier and more extensive treatment than children with IH. In our study, parents perceived a greater impact on QoL for males with ED and suggests that male children with ED may under-report their OHRQoL impact. Finally, this study also highlights the need for clinicians to communicate and educate parents to increase awareness and understanding of the treatment planning process for children with ED and IH.

## Data Availability

The datasets used and analysed during the current study are available from the corresponding author on reasonable request.

## Abbreviations

COHQoL: Child Oral Health Quality of Life Questionnaire
CPQ: Child Perception Questionnaire
DDUH: Dublin Dental University Hospital
ED: Ectodermal Dysplasia
FIS: Family Impact Scale
HSE: Health Service Executive (Ireland’s Public Health System)
IH: Isolated Hypodontia
OHIP: Oral Health Impact Profile
OHRQoL: Oral Health Related Quality of Life
OPG: Orthopantomogram
P-CPQ: Parental-Caregiver Perceptions Questionnaire
QoL: Quality of Life
SPSS: Statistical Package for Social Sciences
JREC: Tallaght University Hospital/St. James's Hospital Joint Research Ethics Committee
TAC: Tooth Agenesis Code
TSA: Tooth-Site Absence
